# Cellular Trafficking of Thymosin Beta-4 in HEPG2 Cells Following Serum Starvation

**DOI:** 10.1371/journal.pone.0067999

**Published:** 2013-08-14

**Authors:** Giuseppina Pichiri, Pierpaolo Coni, Sonia Nemolato, Tiziana Cabras, Mattia Umberto Fanari, Alice Sanna, Eliana Di Felice, Irene Messana, Massimo Castagnola, Gavino Faa

**Affiliations:** 1 Divisione di Anatomia Patologica, Dipartimento di Citomorfologia, University of Cagliari, Cagliari, Italy; 2 Dipartimento di Scienze della Vita e dell'Ambiente, Università di Cagliari, Cagliari, Italy; 3 Istituto di Biochimica e di Biochimica Clinica, Università Cattolica and/or Istituto per la Chimica del Riconoscimento Molecolare, Consiglio Nazionale delle Ricerche, Roma, Italy; Biological Research Centre of the Hungarian Academy of Sciences, Hungary

## Abstract

Thymosin beta-4 (Tβ4) is an ubiquitous multi-functional regenerative peptide, related to many critical biological processes, with a dynamic and flexible conformation which may influence its functions and its subcellular distribution. For these reasons, the intracellular localization and trafficking of Tβ4 is still not completely defined and is still under investigation in in vivo as well as in vitro studies.

In the current study we used HepG2 cells, a human hepatoma cell line; cells growing in normal conditions with fetal bovine serum expressed high levels of Tβ4, restricted to the cytoplasm until 72 h. At 84 h, a diffuse Tβ4 cytoplasmic immunostaining shifted to a focal perinuclear and nuclear reactivity. In the absence of serum, nuclear reactivity was localized in small granules, evenly dispersed throughout the entire nuclear envelop, and was observed as earlier as at 48 h. Cytoplasmic immunostaining for Tβ4 in HepG2 cells under starvation appeared significantly lower at 48 h and decreased progressively at 72 and at 84 h. At these time points, the decrease in cytoplasmic staining was associated with a progressive increase in nuclear reactivity, suggesting a possible translocation of the peptide from the cytoplasm to the nuclear membrane. The normal immunocytochemical pattern was restored when culture cells submitted to starvation for 84 h received a new complete medium for 48 h.

Mass spectrometry analysis, performed on the nuclear and cytosolic fractions of HepG2 growing with and without serum, showed that Tβ4 was detectable only in the cytosolic and not in the intranuclear fraction. These data suggest that Tβ4 is able to translocate from different cytoplasmic domains to the nuclear membrane and back, based on different stress conditions within the cell.

The punctuate pattern of nuclear Tβ4 immunostaining associated with Tβ4 absence in the nucleoplasm suggest that this peptide might be localized in the nuclear pores, where it could regulate the pore permeability.

## Introduction

Thymosin beta-4 (Tβ4) is a naturally occurring peptide, first isolated in 1966 [1], containing 43 amino acid residues [2]. Tβ4 activity has been mainly related to the regulation of actin polymerization in living cells [3], acting as an actin-sequestering peptide in mammalian cells [4]. In recent years, Tβ4 has been proposed as a multi-functional regenerative peptide [5], being involved in many critical biological activities, including angiogenesis [6], wound healing [7], inflammatory response [8] and cell migration and survival [9]. In colon carcinoma cells, over-expression of Tβ4 has been associated with resistance to apoptosis, via down-regulating Fas and up-regulating surviving genes [10]. In another study, it was shown that Tβ4 is able to regulate induced-proinflammatory cytokine, blocking RelA/p65 nuclear translocation [11]. Tβ4 may also have activities independent from the G-actin-binding properties and its dynamic, unstructured and flexible conformation seems to be determinant. [12].

To better understand the role of its small peptide, several studies have analyzed in detail his intracellular localization. In resting macrophages, immunoreactivity for Tβ4 was found to be restricted to the cytoplasm, in the absence of any nuclear immunostaining [13]. Labelled Tβ4 injected into *Xenopus laevis* oocytes was equally distributed between the cytoplasmatic and nuclear compartments [14]. In the human mammary carcinoma MCF-7 cell line, a variable Tβ4 cytoplasmic immunoreactivity, was found constantly associated with an additional nuclear staining [15]. Experiments with microinjection of two fluorescently labeled Tβ4 fragments into HeLa cells supported the hypothesis of the existence of specific active transport mechanisms regulating translocation of this peptide into the cell nucleus [15].

In another study, polyamine depletion in migrating IEC-6 cells induced a translocation of Tß4 into the nucleus [16].

On the contrary, another study using different Tβ4 variants, underlined a possible passive but regulated diffusion that might shuttle this peptide into the nucleus, suggesting that Tβ4 translocation could be regulated by the change of the pore permeability [17].

Recently, Tβ4 has been reported to be expressed in high levels in normal and neoplastic hepatocytes [18]. On the basis of these data, it seemed of some interest to study the immunoreactivity of Tβ4 in HepG2 cells, a human hepatoma cell line with a strong Tβ4 immunocytochemistry expression, frequently used as an in vitro model to investigate the regulation of hepatocytes cells growth. [19]–[22]. In the current study, HepG2 cells were cultured with complete medium or without fetal bovine serum, in order to better analyze the Tβ4 expression pattern and Tβ4 localization during different environmental conditions.

## Materials and Methods

### Cell culture

Commercial human cell line HepG2 (ICLC HTL95005), were obtained from the Istituto Nazionale per la Ricerca sul Cancro c/o CBA (ICLC, Genova). The culture medium used for this purpose was a mixture of MEM (EBSS), 10% fetal bovine serum (FBS), 100 units/ml penicillin, 100 µg/ml streptomycin, 2 mM L-Glutamine, 1% non-essential amino acids. To perform different experimental conditions, confluent cells were isolated using trypsin/EDTA and, for the experimental procedure, samples of 2–3×10^4^ cells/cm^2^ HepG2 cells were plated on different glass coverslips at 37°C, 5% CO_2_. After 24 h of growth with complete medium, cells were cultured with complete culture medium or with medium without FBS for 48 h, 72 h and 84 h. In cells submitted to serum starvation for 84 h complete medium with FBS was added and cells were analyzed at 24 and 48 h. All samples were washed with PBS and fixed with acetone for 20 min, air dried for 30 min and then stored at −20°C.

### Tβ4 immunocytochemistry

Tβ4 immunocytochemistry was performed as previously reported in human liver biopsies [18]. Briefly, cells were rehydrated, and endogenous peroxidase activity was quenched (3 min) by 0.3% hydrogen peroxide in methanol. Cells were incubated with 10% normal goat serum in phosphate buffered saline (PBS) for 5 min to block non-specific binding and then incubated (30 min) with a polyclonal anti-Tß4 antibody (Bachem-Peninsula Lab, San Carlos, CA, USA) diluted 1∶100 in a blocking solution. Cells were extensively washed with PBS containing 0.01% Triton X-100 and incubated with a secondary reagent (Envision kit) according to the manufacturer's instructions (Dako, Glostrup, Denmark). After additional washes, color was developed using AEC reagent (Dako, Glostrup, Denmark); cells were counterstained with hematoxylin and mounted.

### Cell fractionation

The modified method of Galan et al [23] was applied for cell fractionation. Briefly, cells (4×10^7^ cells/mL) were incubated for 15 min in 2 mL of ice-cold buffer A (10 mM HEPES pH 7.9, 10 mM KCl, 0,1 mM EDTA, 0.1 mM EGTA, 1 mM DTT and Complete Protease Inhibitor Cocktail (Roche, Basel, Switzerland), 1 tablet/10 mL) and then shaken vigorously for 2 min before adding β-octyl-glucopyranoside (0.1% final concentration). The cytosolic fraction was separated from nuclei by centrifugation at 1300×g, 4°C, for 5 min, and stored at −80°C. The nuclear pellet was washed twice in buffer A and then resuspended in buffer B (20 mM HEPES pH 7.9, 0.4 mM NaCl, 1.0 mM EDTA, 1.0 mM EGTA, 1 mM DTT and 1 tablet/10 mL of Complete Protease Inhibitor Cocktail), rotated at 4°C for 15 min and then centrifuged at 1000×g for 5 min. The supernatant (nuclear fraction) was collected and stored at −80°C.

### Treatment of cytosolic and nuclear fractions and RP-HPLC-ESI-MS analysis

Cytosolic and nuclear proteins/peptides were fractionated (30 KDa cut-off) with Amicon centrifugal filter units (Millipore, Billerica, MA, USA), dialyzed in 25 mM sodium acetate pH 4.2 and then lyophilized. The lyophilized powder was dissolved in 120 µL of 0.1% aqueous trifluoroacetic acid (TFA), and the solution centrifuged at 8000×g for 10 min at 4°C. 100 µL of the supernatant was injected in the RP-HPLC-ESI-MS apparatus.

HPLC-ESI-MS measurements were carried out by a Surveyor HPLC system (ThermoFisher, San Jose, CA, USA) connected by a T splitter to a photodiode-array detector and an LCQ Advantage mass spectrometer (ThermoFisher). The chromatographic column was a Vydac (Hesperia, CA, USA) C8 with 5 µm particle diameter (column dimensions 150×2.1 mm). The following solutions were utilized for the chromatographic separations: (eluent A) 0.056% aqueous TFA and (eluent B) 0.050% TFA in acetonitrile-water 80/20 (v/v). The applied gradient was linear from 0 to 54% in 39 min (linear) and from 54% to 100% in 10 min (linear), at a flow rate of 0.30 mL/min. The T splitter permitted 0.20 mL/min to flow toward the diode array detector and 0.10 mL/min toward the ESI source. The photodiode array detector was set at a wavelength of 214 and 276 nm. During the first 5 min of separation eluate was not analized by the mass spectrometer in order to avoid source contamination and instrument damage due to the high salt concentration. Mass spectra were collected every 3 millisecond in the positive ion mode. MS spray voltage was 5.0 kV and capillary temperature was 255°C.

### HPLC-ESI-MS data analysis and quantification

The experimental average mass value of Thymosin β4, previously characterized and previously reported by our laboratory [24], was obtained by deconvolution of ESI-MS spectra automatically performed using MagTran 1.0 software [25] and compared with the theoretical mass value, available at the Swiss-Prot Data Bank (http://us.expasy.org/tools) with the accession code P62328.

Quantification was based on the area of the RP-HPLC-ESI-MS eXtracted Ion Current (XIC) peak, which is proportional to the peptide/protein concentration under constant analytical conditions [26]. The ions used to quantify the Tβ4 were: 993.80 (+5), 1241.90 (+4) and 1655.50 (+3) m/z. A window of ±0.5 Da was used to extract ion chromatograms. The following post-translational modifications of the Tβ4 were also searched in the chromatogram: Tβ4 sulfoxide, Tβ4 acetylated at the level of lateral chains, Tβ4 phosphorylated.

## Results

### Immunocytochemistry expression

Tβ4 immunocytochemistry of HepG2 cells growing in normal conditions and after starvation was performed at 48 h, 72 h and 84 h.

**Figure 1:**
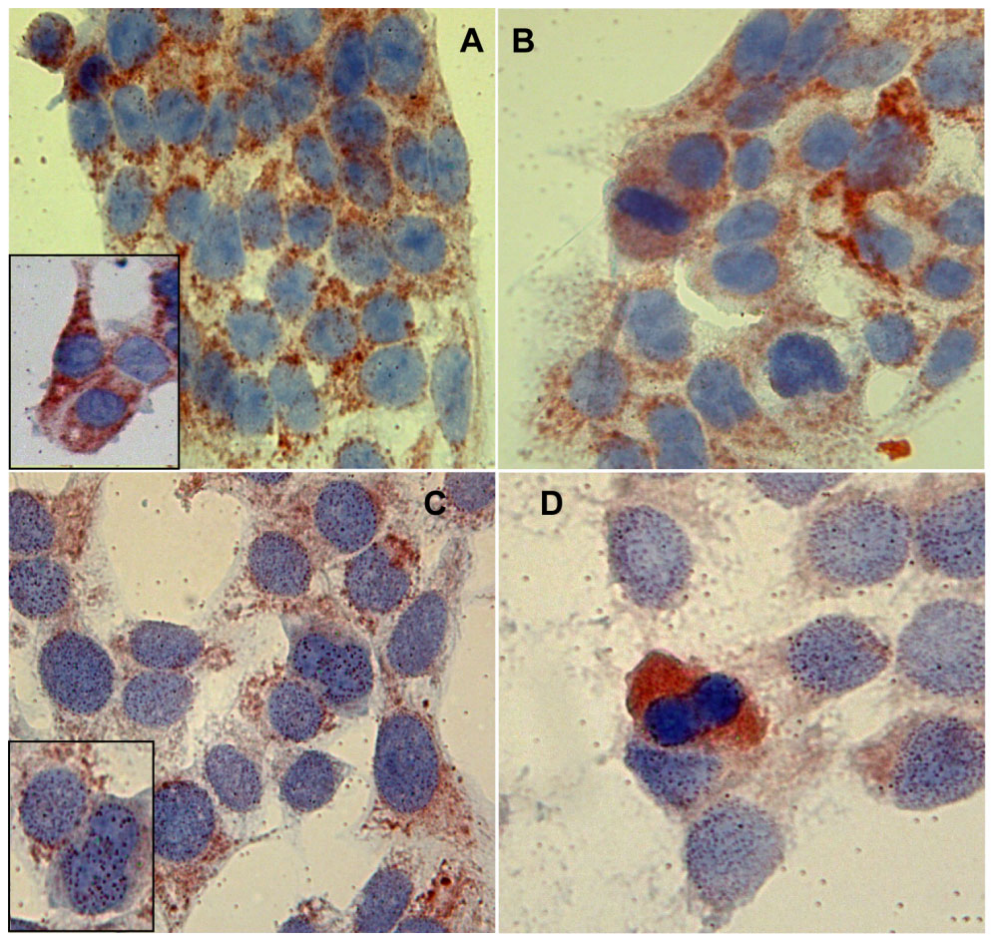
Thymosin β 4 immunoreactivity after 48 h of starvation. Tβ 4 immunoreactivity in HepG2 cells cultured for 48 h with serum (A and B, magnification 1000×) and without serum (C and D, magnification 1000×). Inserts in A and in C represent one particular of the intranuclear reactivity of the respective pictures (magnification 1000×).

HepG2 cells growing in complete medium for 48 h showed high levels of Tβ4 expression. Immunoreactivity for the peptide was detected in the cytoplasm of the vast majority of culture cells, appearing as small granules probably reflecting localization of the peptide in cytoplasmic vacuoles (Fig. 1a). Tβ4 expression was evenly distributed throughout each single sample. No significant differences, regarding the degree of immunostaining for Tβ4 were observed in HepG2 cells growing with complete medium (Fig. 1a, 1b). At high power, reactivity for the peptide was restricted to the cytoplasm of culture cells, in the absence of any nuclear immunostaining (Fig. 1b). The pattern of immunoreactivity for Tβ4 changed in cells undergoing mitosis, being characterized by a diffuse homogeneous cytoplasmic staining and in the absence of any granular reactivity (Fig. 1b).

On the contrary, HepG2 cells growing for 48 h in the absence of fetal bovine serum were characterized by an apparent reduction in the degree of Tβ4 expression. The peptide was mainly detected in the cytoplasm of culture cells in a granular pattern, but the intensity of immunostaining was significantly lower as compared to HepG2 cells growing in complete medium (Fig. 1c). Moreover, under starvation, immunoreactivity for Tβ4 was also detected at the nuclear level: nuclear staining for the peptide was localized in granules organized as small roundish spots, evenly dispersed only throughout the entire nuclear envelop (Fig. 1c, 1d). The peculiar pattern of Tβ4 reactivity previously described in mitotic figures was confirmed even in cells growing in the absence of FBS: in mitotic cells immunoreactivity for Tβ4 was restricted to the cytoplasm with a homogeneous staining pattern (Fig. 1d).

**Figure 2:**
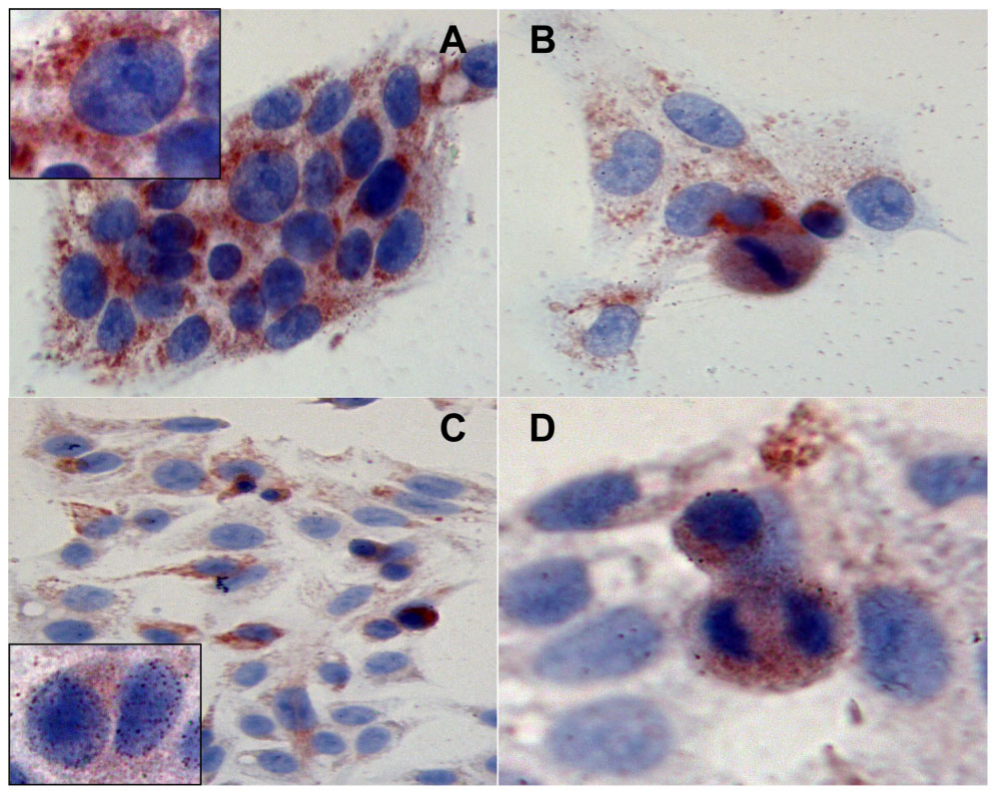
Thymosin β 4 immunoreactivity after 72 h of starvation. Tβ 4 immunoreactivity in HepG2 cells cultured for 72 h with serum (A and B, magnification 1000×) and without serum (C and D, magnification respectively 400 and 1000×). Inserts in A and in C represent one particular of the intranuclear reactivity of the respective pictures (magnification 1000×).

At this time point, HepG2 cells growing for 72 h in complete medium were characterized by a higher immunoreactivity for Tβ4 (Fig. 2a). Staining for the peptide was observed in the vast majority of cells, always restricted to the cytoplasm. Fine and coarse Tβ4-reactive granules were observed scattered throughout the whole cytoplasm. At high power, cells in mitosis showed, even at this time point, an homogeneous cytoplasmic reactivity (Fig. 2b).

Occasionally, cells showed a different pattern of reactivity for Tβ4, characterized by a perinuclear spot, suggestive for a localization of the peptide in the trans-Golgi network (Fig. 2b).

Marked differences in the immunocytochemical pattern were detected at 72 h in culture cells under serum deprivation. The degree of immunoreactivity appeared much lower, as compared to cells growing with a complete medium. Moreover, immunostaining for Tβ4 was unevenly distributed, with positive cells intermingled with negative cells (Fig. 2c). Cells undergoing apoptosis, morphologically characterized by cell shrinkage and chromatin condensation, were characterized by a strong homogeneous cytoplasmic staining, probably reflecting cell shrinkage (Fig. 2c). Even at nuclear level, reactivity for Tβ4 was uneven: some cells showed a strong nuclear staining (Fig. 2c) appearing as multiple roundish spots diffuse to the entire nuclear envelope. Other cells did not show any nuclear reactivity for the peptide (Fig. 2d). Marked differences regarding Tβ4 expression were also found, at this time point, in mitotic cells: the homogeneous pattern previously described was substituted by a granular immunoreactivity, always restricted to the cytoplasm (Fig. 2d).

**Figure 3:**
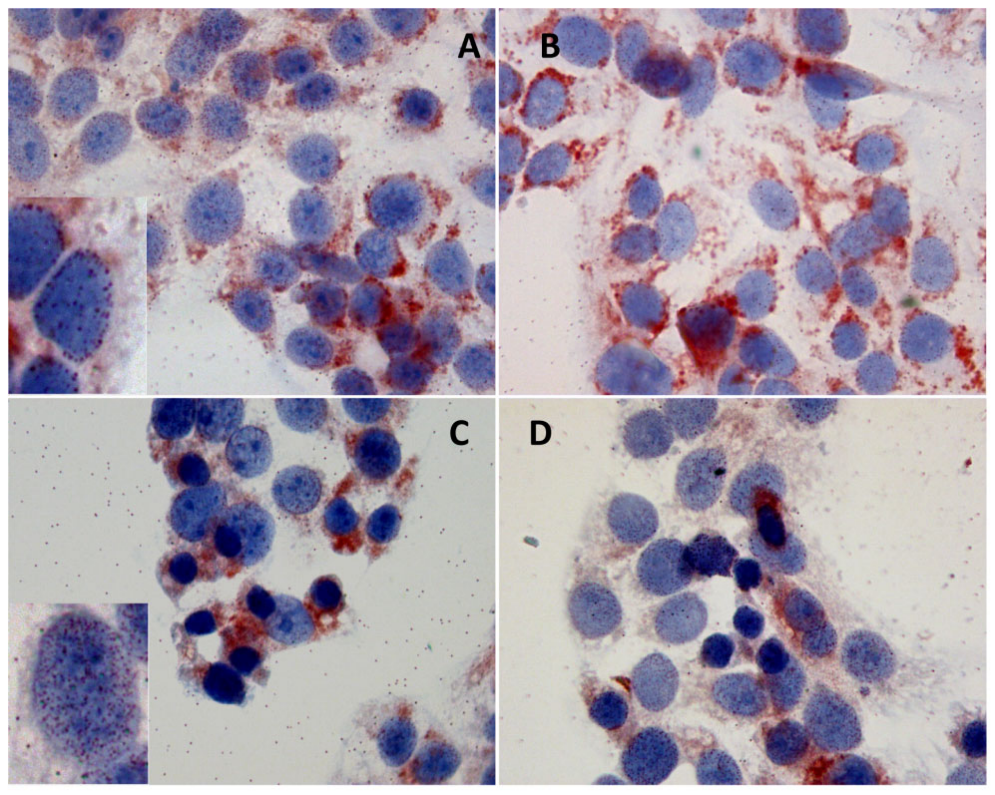
Thymosin β 4 immunoreactivity after 84 h of starvation. Tβ 4 immunoreactivity in HepG2 cells cultured for 84 h with serum (A and B, magnification 1000×) and without serum (C and D, magnification 1000×). Inserts in A and in C represent one particular of the intranuclear reactivity of the respective pictures (magnification 1000×).

Immunoreactivity for Tβ4 changed significantly in cells growing in complete medium for 84 h. First of all, we observed a decrease in cytoplasmic immunostaining for the peptide, associated with its uneven distribution (Fig. 3a). Moreover, we detected marked changes in the localization of Tβ4, which appeared to be mainly localized in a perinuclear spot (Fig. 3a, 3b). At this time point, for the first time, we observed immunoreactivity for Tβ4 in the nuclear envelop in cells growing in complete medium (Fig. 3a, insert). This nuclear immunoreactivity was detected in a minority of culture cells, whereas the vast majority of HepG2 cells showed a predominant perinuclear cytoplasmic immunostaining (Fig. 3b).

After 84 h of serum deprivation, the highest levels of reactivity for Tß4 were observed in the cytoplasm of cells undergoing apoptosis, contrasting with a weak immunostaining in the surviving cells (Fig. 3c, 3d). The marked decrease in cytoplasmic immunostaining for Tß4 was paralleled by the increase in nuclear reactivity, which was localized, as previously described, in the nuclear envelope (Fig. 3c, insert).

**Figure 4:**
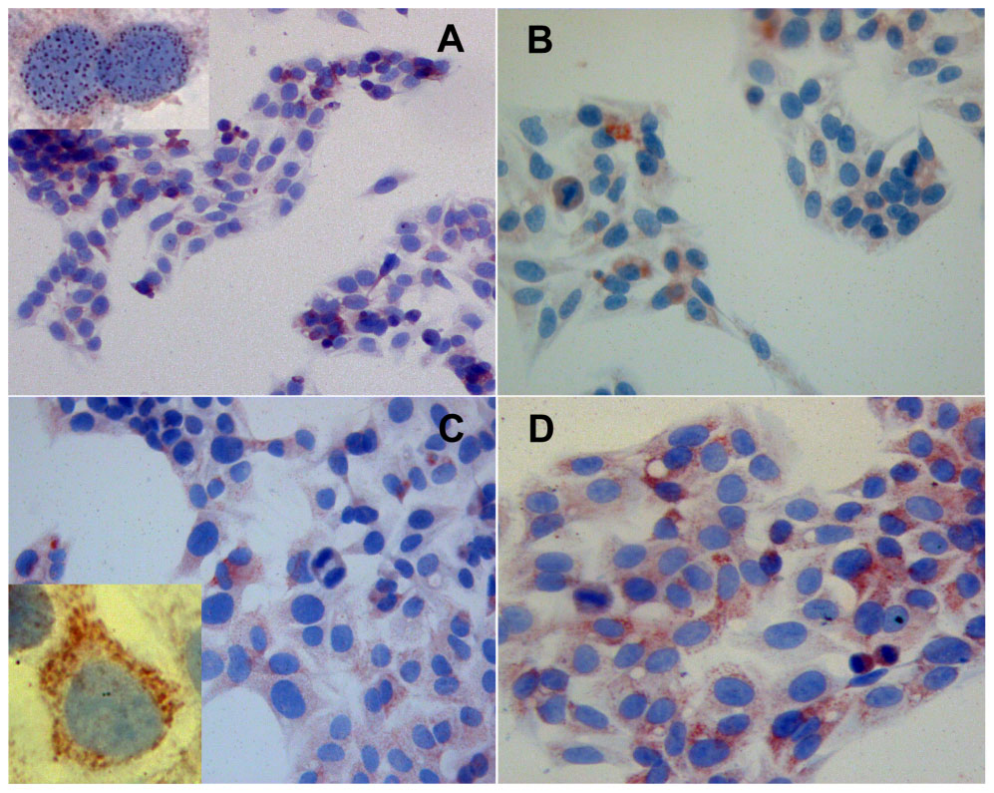
Thymosin β 4 immunoreactivity 24 and 48 h with complete medium after starvation. Tβ 4 immunoreactivity in HepG2 cells cultured for 84 h without serum and shifted in complete medium for 24 h (A and B, magnification 400×) and for 48 h (C and D, magnification 400×). Inserts in A and in C represent one particular of the intranuclear reactivity of the respective pictures (magnification 1000×).

When the medium was removed in cells submitted to starvation for 84 h, and a new complete medium with serum was added, 24 h later we detected significant changes in Tβ4 immunoreactivity (Fig. 4a, 4b). At this time point, the peptide appeared evenly distributed in the cytoplasm of the vast majority of cells (Fig. 4a). After serum implementation, mitotic cells with a weak homogeneous immunoreactivity in the entire cytoplasm increased. (Fig. 4b). Tβ4 immunostaining, at this time point was also detected in the nuclear envelope of a large number of culture cells (Fig. 4a, insert).

A progressive increase in immunostaining for Tβ4 was evident in cells 48 h after introducing fresh medium. At this time point, the peptide appeared evenly distributed, immunoreactivity being mainly localized in the cytoplasm of the vast majority of cells (Fig. 4c). At higher power, marked changes were detected regarding the subcellular localization of Tβ4: the peptide moved from the nucleus towards the cytoplasm, leaving the nucleus devoid of Tβ4 (Fig. 4c, insert).

### Mass spectrometry analysis

**Figure 5:**
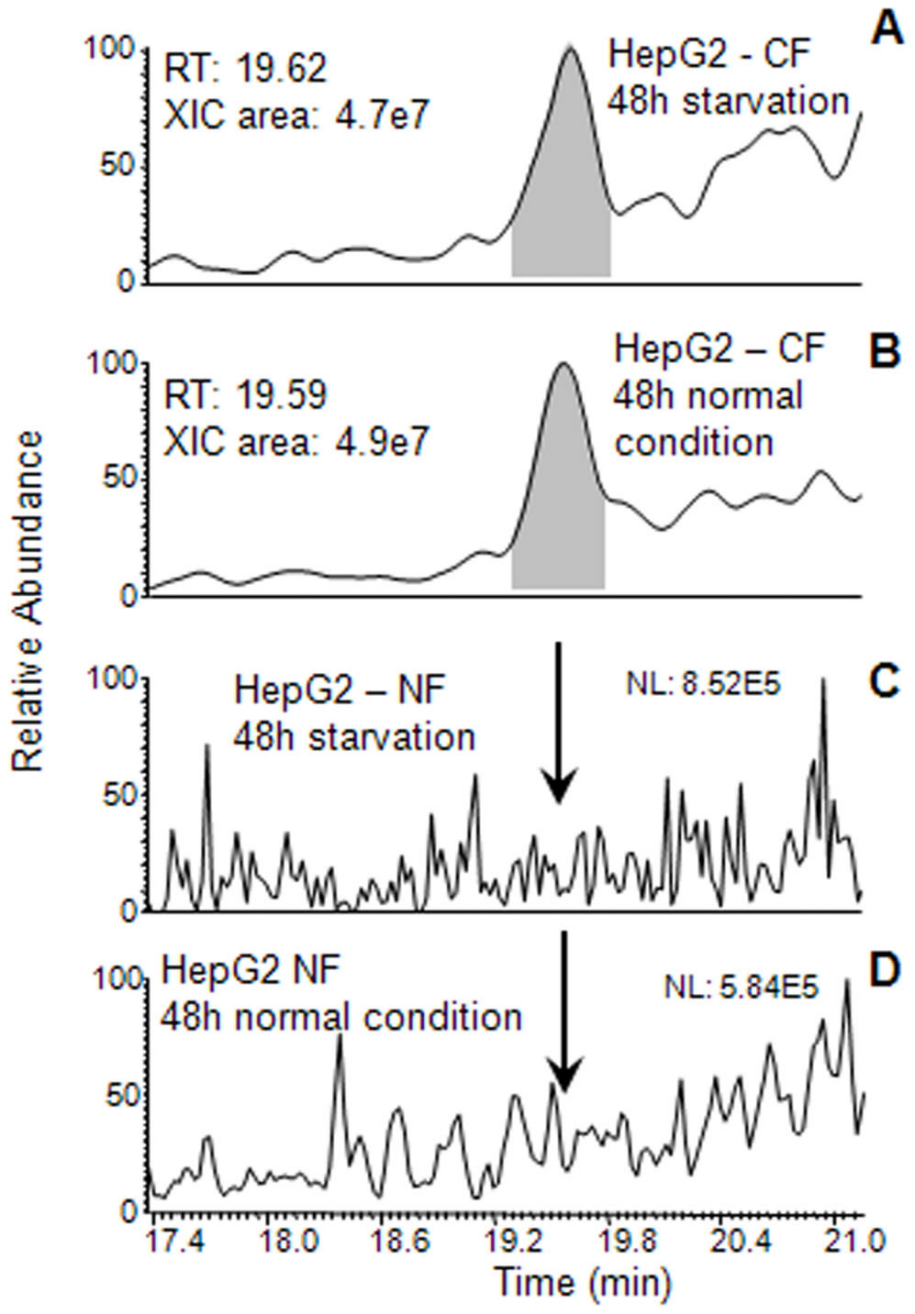
XIC peak of Tβ4 evidenced by HPLC-ESI-MS analysis of the cytosolic (CF) and nuclear fractions (NF) of HepG2 cells grown for 48 h. Enlargements of the chromatographic profiles in the range 17.4–21.1 min are reported. Tβ4 search in CF of the cells grown under starvation and normal conditions, panel A and panel B; Tβ4 search in NF of the cells grown under starvation and normal conditions, panel C and panel D; arrows indicate the absence of the Tβ4 in its chromatographic elution range.

The cytosolic and nuclear fractions of HepG2 cells grown for 48 h under normal conditions and after starvation were analyzed by HPLC-ESI-MS. Tβ4 was detected only in the cytosolic fractions (CF). The chromatographic position (19.3–19.8 min), the experimental average mass value (4963.2±0.5 Da) and MS/MS spectrum perfectly corresponded with the data registered on a standard sample of Tβ4. The Fig. 5 reports results of XIC search of Tβ4 in the cytosolic and nuclear fractions (NF) under the two experimental conditions. The level of Tβ4 was not different in normal and stressed conditions CFs. The known post-translational modified derivatives of Tβ4 were not detected in any samples.

## Discussion

The data presented in this study demonstrate that HepG2 cells normally express high levels of Tβ4, which may be easily identifiable by immunocytochemistry. As a consequence, HepG2 cells may be considered a reliable and useful model for the study of Tβ4 expression in human hepatocytes in different environmental conditions, and should be added to the list of Tβ4-producing cell lines previously reported by Hannappel et al [27].

Our study reinforces the hypothesis on the “enigma” [28] of beta- thymosins, by adding new interesting peculiar features to this fascinating peptide: its ability to change conformation and to translocate from one cytoplasmic domain to another, and from the cytoplasm to the nucleus and back, according with different environmental stimuli. In physiological situations, here represented by culture cells growing in fresh complete medium, Tβ4 was always restricted to the cytoplasm, showing a granular pattern highly suggestive for its localization in cytoplasmic vacuoles. This immunostaining has been observed without significant changes for 72 h, in the complete absence of any nuclear immunoreactivity. A similar immunohistochemical pattern has been recently reported by our group in the normal human liver: in that study, the hepatocytes showed a strong granular immunoreactivity for Tβ4, which was stored in vacuoles irregularly dispersed throughout the entire cytoplasm [18].

At 84 h, a time point in which culture cells become under stress due to scarcity of nutrients and accumulation of waste metabolic products, significant modifications in the Tβ4 subcellular localization were found. The previously reported diffuse cytoplasmic immunostaining was substituted by a focal reactivity, mainly represented by a perinuclear spot-like staining pattern highly suggestive of a translocation of Tβ4 from vacuoles towards the trans-Golgi network. A similar cytoplasmic localization of Tβ4 has been previously observed by our group in the human normal colon mucosa, in the proximity of colon cancer [29]. In that study, Tβ4-immunoreactive perinuclear spots were frequently detected in the enterocytes covering the intestinal mucosa in close proximity of colon cancer. In the same study, this peculiar immunohistochemical pattern was also found in cells showing the morphological changes typical of colon dysplasia. These findings suggest that Tβ4 translocation from one cytoplasmic compartment to another could play a possible role in colon carcinogenesis. The significance of Tβ4 translocation to the perinuclear regions, putatively to the trans-Golgi network, remains at the best of our knowledge unknown. Our data confirm that Tβ4 may change its subcellular localization in different environmental conditions. In this study, the perinuclear spot-like pattern of immunoreactivity was exclusively found in cells under stress, here represented by cells growing in a medium without serum, or in cells living more than three days in complete medium. Taken together, these data indicate cytoplasmic translocation from cytoplasmic vacuoles towards the perinuclear regions as a defense mechanism of HepG2 cells against environmental changes causing cell stress. The intimate mechanism by which Tβ4 reaches the trans-Golgi network, given the reported absence of a signal peptide in this protein, remains unknown [30].

The second most important finding of this work is the nuclear reactivity of the peptide, appearing as multiple Tβ4-reactive roundish spots dispersed throughout the entire nuclear envelop. This phenomenon was observed late, at 84 h, in culture cells growing in complete medium, and much earlier, at 48 h, in cells growing in the absence of serum. The nuclear positivity seems to be related to the starvation-induced stress; in fact, when the old complete medium was substituted with a fresh one, and serum-deprived cells received the entire medium, the “normal” immunocytochemical cytoplasmic pattern was restored, and the peptide was not anymore detected at the nuclear level.

The ability of Tβ4 to cross the nuclear membrane was first reported in a study in which the peptide was injected in *Xenopus laevis* oocytes, reaching both nuclear and cytoplasmic compartments [14]. The ability of Tβ4 to change its localization according to different external stimuli was also described in IEC-6 cells. In untreated control cells, the peptide was restricted to the cytoplasm, whereas following cell depletion of polyamines, cytoplasmic expression decreased, paralleled by the appearance of nuclear staining for Tβ4 [16]. Nuclear localization of Tβ4, always associated with its cytoplasmic expression, has been reported in another cell line, the human mammary carcinoma MCF-7 [15]. In that study, authors suggested that Tβ4 might be shuttled into the nucleus utilizing an active transport mechanism, requiring an unidentified soluble cytoplasmic factor. Recently, this factor has been reported to be identified in the human MLH1, a key enzyme of DNA mismatch repair with several additional functions, including the intranuclear transport of Tβ4 [31].

On the other hand, a passive but regulated diffusion has been proposed as the mechanism that could explain the ability of Tβ4 to shuttle into the nucleus [17]. According to this study, the Tβ4 cytoplasmic-nuclear diffusion might be due to changes in the barrier function of the nuclear pores [17].

Regarding the complex trafficking of the Tβ4 from the cytoplasm towards the nucleus here we provide new findings: based on our immunocytochemical and biochemical data. Nuclear translocation of Tβ4 seems to be localized in the nuclear envelope and not inside of the nucleus.

Mass spectrometry analysis of the nuclear proteins fraction seems to confirm that Tβ4 is not detectable inside of the nucleus and, if present, it is under the detection limits of the HPLC-ESI-MS technique in use. This finding is confirmed by the peculiar immunocytochemical pattern of nuclear immunostaining, characterized by a punctuated reactivity, highly suggestive of a Tβ4 localization in the nuclear pores.

Regarding the significance of nuclear translocation of Tβ4, it has been suggested that this actin-binding peptide could function as a modulator of gene transcription [32]. Interestingly, it was recently suggested that Tβ4 may be involved in the bidirectional movement of specific proteins between nucleus and cytoplasm. In that study, Tβ4 has been shown to regulate nuclear translocation of multiple proteins with a consequent modulation of the inflammatory response [11].

The pattern of nuclear reactivity for Tβ4 here reported confirm this hypothesis, and suggest that Tβ4 localization in nuclear pores could play a relevant role in the regulation of the barrier function of nuclear pores [17].

Another interesting finding of our study is represented by the different pattern of Tβ4 immunoreactivity in mitotic cells as compared to culture cells in a resting phase. Cells undergoing mitosis showed a predominant homogeneous staining pattern diffuse to the entire cytoplasm, contrasting with the granular or with the spot-like immunostaining detected in resting cells. The marked differences in Tβ4 staining pattern in mitotic cells could indicate a translocation of the peptide from vacuoles towards the cytoskeleton. Further studies on Tβ4 subcellular localization at the ultramicroscopic level might shed light on the intimate significance of these lasts observations.

Taken all together, these data clearly show the ability of Tβ4 to move from one cytoplasmic domain to the another, and to shuttle from the cytoplasm towards the nuclear membrane and back, depending on changes of environmental conditions. Further studies are needed in order to better explain the intimate molecular mechanisms that are at the basis of this translocation.

## References

[1] Goldstein AL, Slater FD, White A (1966) Preparation, assay, and partial purification of a thymic lymphocytopoietic factor (thymosin). Proc Natl Acad Sci USA 56: 1010–1007. 5230175 10.1073/pnas.56.3.1010 PMC219962

[2] Low TL, Hu SK, Goldstein AL (1981) Complete amino acid sequence of bovine thymosin beta 4: a thymic hormone that induces terminal deoxynucleotidyl transferase activity in thymocyte populations. Proc Natl Acad Sci USA 78: 1162–1166. 6940133 10.1073/pnas.78.2.1162 PMC319967

[3] Ballweber E, Hannappel E, Huff T, Stephan H, Haener M, et al (2002) Polymerization of chemically cross-linked actin:thymosin beta(4) complex to filamentous actin: alteration in helical parameters and visualization of thymosin beta(4) binding on F-actin. J Mol Biol 315: 613–625. 11812134 10.1006/jmbi.2001.5281

[4] Sanders MC, Goldstein AL, Wang YL (1992) Thymosin beta 4 (Fx peptide) is a potent regulator of actin polymerization in living cells. Proc Natl Acad Sci USA 89: 4678–4682. 1584803 10.1073/pnas.89.10.4678 PMC49146

[5] Goldstein AL, Hannappel E, Sosne G, Kleinman HK (2012) Thymosin β4: a multi-functional regenerative peptide. Basic properties and clinical applications. Expert Opin Biol Ther 12: 37–51. 22074294 10.1517/14712598.2012.634793

[6] Koutrafouri V, Leondiadis L, Avgoustakis K, Livaniou E, Czarnecki J, et al (2001) Effect of thymosin peptides on the chick chorioallantoic membrane angiogenesis model. Biochim Biophys Acta 1568: 60–66. 11731086 10.1016/s0304-4165(01)00200-8

[7] Malinda KM, Sidhu GS, Mani H, Banaudha K, Maheshwari RK, et al (1999) Thymosin beta 4 accelerates wound healing. J Invest Dermatol 113: 364–368. 10469335 10.1046/j.1523-1747.1999.00708.x

[8] Badamchian M, Fagarasan MO, Danner RL, Suffredini AF, Damavandy H, et al (2003) Thymosin β 4 reduces lethality and downmodulates inflammatory mediators in endotoxin induced septic shock. Int Immunopharmacol 3: 1225–1233. 12860178 10.1016/S1567-5769(03)00024-9

[9] Bock-Marquette I, Saxena A, White MD, DiMaio JM, Srivasta D (2004) Thymosin beta 4 activates integrin-linked kinase and promotes cardiac cell migration, survival and cardiac repair. Nature 432: 466–472. 15565145 10.1038/nature03000

[10] Hsiao HL, Wang WS, Chen PM, Su Y (2006) Overexpression of thymosin beta-4 renders SW480 colon carcinoma cells more resistant to apoptosis triggered by FasL and two topoisomerase II inhibitors via down-regulating Fas and up-regulating Survivin expression, respectively. Carcinogenesis 27: 936–944. 16364925 10.1093/carcin/bgi316

[11] Qiu P, Wheater MK, Qiu Y, Sosne G (2011) Thymosin beta4 inhibits TNF-alpha-induced NF-kappa B activation, IL-8 expression, and the sensitizing effects by its partners PINCH-1 and ILK. FASEB J 25: 1815–26. 21343177 10.1096/fj.10-167940 PMC3101037

[12] David C, Nabila T, Christian A, Janet A (2010) Thymosin β4: structure, function, and biological properties supporting current and future clinical applications. Ann. NY Acad Sci 1194: 179–189. 10.1111/j.1749-6632.2010.05492.x 20536467

[13] Yu FX, Lin SC, Morrison-Bogorad M, Yin HL (1994) Effects of thymosin β4 and thymosin β10 on actin structures in living cells. Cell Motil Cytoskeleton 27: 13–25. 8194107 10.1002/cm.970270103

[14] Watts JD, Cary PD, Sautiere P, Crane-Robinson C (1990) Thymosins: both nuclear and cytoplasmic proteins. Eur J Biochem 192: 643–651. 2209614 10.1111/j.1432-1033.1990.tb19271.x

[15] Huff T, Rosorius O, Otto AM, Müller CSG, Ballweber E, et al (2004) Nuclear localization of the G-actin sequestering peptide thymosin β4. Journal of Cell Science 117: 5333–5343. 15466884 10.1242/jcs.01404

[16] McCormack SA, Ray RM, Blanner PM, Johnson LR (1999) Polyamine depletion alters the relationship of F-actin, G-actin, and thymosin β4 in migrating IEC-6 cells. Am J Physiol 276: C459–C468. 9950774 10.1152/ajpcell.1999.276.2.C459

[17] Zoubek RE, Hannappel E (2007) Subcellular distribution of thymosin beta4. Ann N Y Acad Sci 1112: 442–450. 17567947 10.1196/annals.1415.031

[18] Nemolato S, Van Eyken P, Cabras T, Cau F, Fanari MU, et al (2011) Expression pattern of thymosin beta 4 in the adult human liver. Eur J Histochem 55: 131–135. 10.4081/ejh.2011.e25 PMC3203477 22073372

[19] Kang S, Song J, Kang H, Kim S, Lee Y, et al (2003) Insulin can block apoptosis by decreasing oxidative stress via phosphatidylinositol 3-kinase- and extracellular signal-regulated protein kinase-dependent signaling pathways in HepG2 cells. Eur J Endocrinol 148: 147–155. 12534368 10.1530/eje.0.1480147

[20] Schamberger CJ, Gerner C, Cerni C (2005) Caspase-9 plays a marginal role in serum starvation-induced apoptosis. Exp Cell Res 302: 115–128. 15541731 10.1016/j.yexcr.2004.08.026

[21] Zhuge J, Cederbaum AI (2006) Serum deprivation-induced HepG2 cell death is potentiated by CYP2E1. Free Radic Biol Med 40: 63–74. 16337880 10.1016/j.freeradbiomed.2005.08.012

[22] Bai J, Cederbaum AI (2006) Cycloheximide protects HepG2 cells from serum withdrawal induced apoptosis by decreasing p53 and phosphorylated p53 levels. J Pharmacol and Experimental Therap 319: 1435–1443. 10.1124/jpet.106.110007 16971506

[23] Galan JA, Paris LL, Zhang H, Adler J, Geahlen RL, et al (2011) Proteomic Studies of Syk-Interacting Proteins Using a Novel Amine-Specific Isotope Tag and GFP Nanotrap. J Am Soc Mass Spectrom 22: 319Y328. 21472591 10.1007/s13361-010-0030-7 PMC3074380

[24] Inzitari R, Cabras T, Pisano E, Fanali C, Manconi B, et al (2009) HPLC-ESI-MS analysis of oral human fluids reveals that gingival crevicular fluid is the main source of oral thymosins beta(4) and beta(10). J Sep Sci 32: 57–63. 19035385 10.1002/jssc.200800496

[25] Zhang Z, Marshall AG (1998) A universal algorithm for fast and automated charge state deconvolution of electrospray mass-to-charge ratio spectra. J Am Soc Mass Spectrom 9: 225–233. 9879360 10.1016/S1044-0305(97)00284-5

[26] Ong SE, Mann M (2005) Mass spectrometry-based proteomics turns quantitative. Nat Chem Biol 1: 252–262. 16408053 10.1038/nchembio736

[27] Hannappel E, Leibold W (1985) Biosynthesis rates and content of thymosin β4 in cell lines. Arch Biochem Biophys 240: 236–241. 2990345 10.1016/0003-9861(85)90028-1

[28] Su HQ, Yin HL (2007) The beta-thymosin enigma. Ann N Y Acad Sci 1112: 45–55. 17495248 10.1196/annals.1415.021

[29] Nemolato S, Restivo A, Cabras T, Coni P, Zorcolo L, et al (2012) Thymosin β4 in colorectal cancer is localized predominantly at the invasion front in tumor cells undergoing epithelial mesenchymal transition. Cancer Biol Ther 13: 191–197. 22233609 10.4161/cbt.13.4.18691

[30] Wodnar-Filipowicz A, Gubler U, Furuichi Y, Richardson M, Horecker BL (1984) Cloning and sequence analysis of cDNA for rat spleen thymosin beta 4. Proc Natl Acad Sci USA 81: 2295–2297. 6201851 10.1073/pnas.81.8.2295 PMC345045

[31] Brieger A, Plotz G, Zeuzem S, Trojan J (2007) Thymosin beta 4 expression and nuclear transport are regulated by hMLH1. Biochem Biophys Res Commun 364: 731–736. 17967441 10.1016/j.bbrc.2007.10.010

[32] Gettemans J, Van Impe K, Delanote V, Hubert T, Vandekerckhove J, et al (2005) Nuclear actin-binding proteins as modulators of gene transcription. Traffic 6: 847–857. 16138899 10.1111/j.1600-0854.2005.00326.x

